# Supplementary material for: Diverse infection models demonstrate robust resistance of Mycobacterium tuberculosis to innate immunity
Source: bioRxiv. 2026 Jun 11:2025.11.20.687493. Preprint. [Version 3] doi: 10.1101/2025.11.20.687493 (PMC13278070; doi:10.1101/2025.11.20.687493)
Supplement: Supplementary file 1 [file NIHPP2025.11.20.687493v3-supplement-1.pdf]

bioRxiv preprint doi: https://doi.org/10.1101/2025.11.20.687493; this version posted June 11, 2026. The copyright holder for this preprint (which was not certified by peer review) is the author/funder, who has granted bioRxiv a license to display the preprint in perpetuity. It is made available under a CC-BY 4.0 International license.

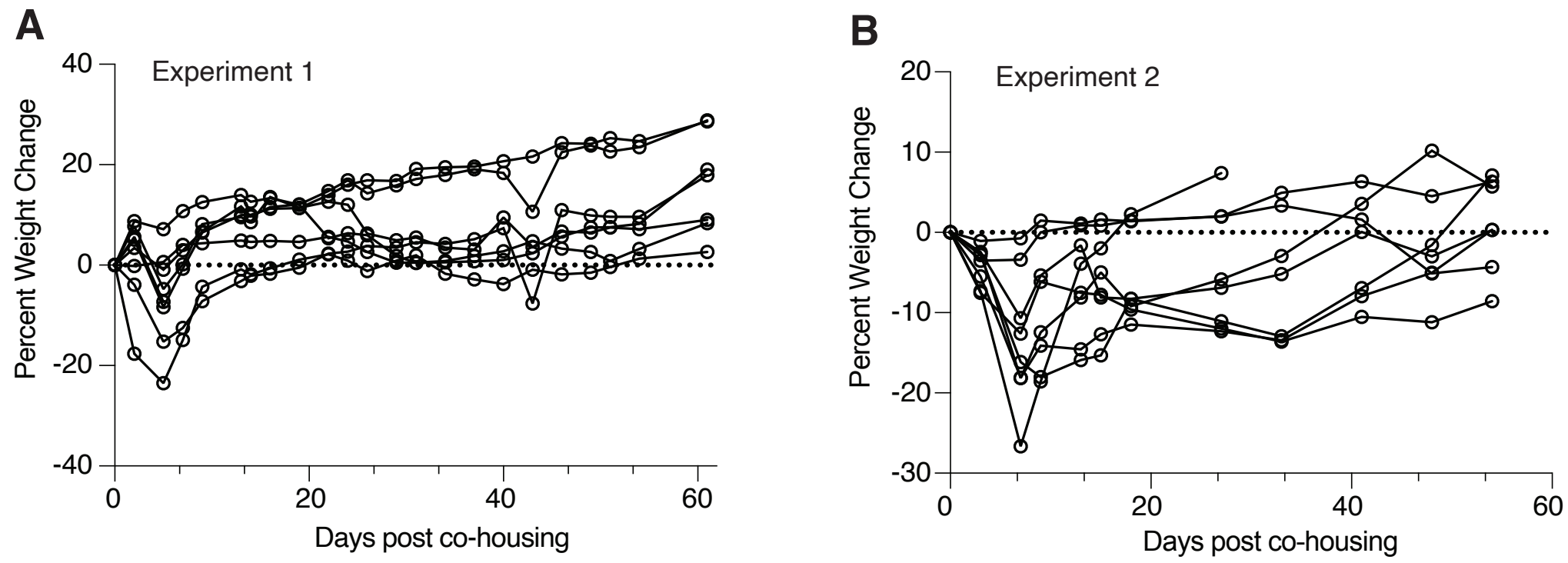

Supplementary Figure 1. Weight changes in co-housed mice.
(**A-B**) Average percent weight change of C57BL/6 mice cohoused with pet shop mice in two individual experiments. Each line represents the average weight change of up to 8 mice in one cage. Mice were weighed every 3-7 days over 60 days.

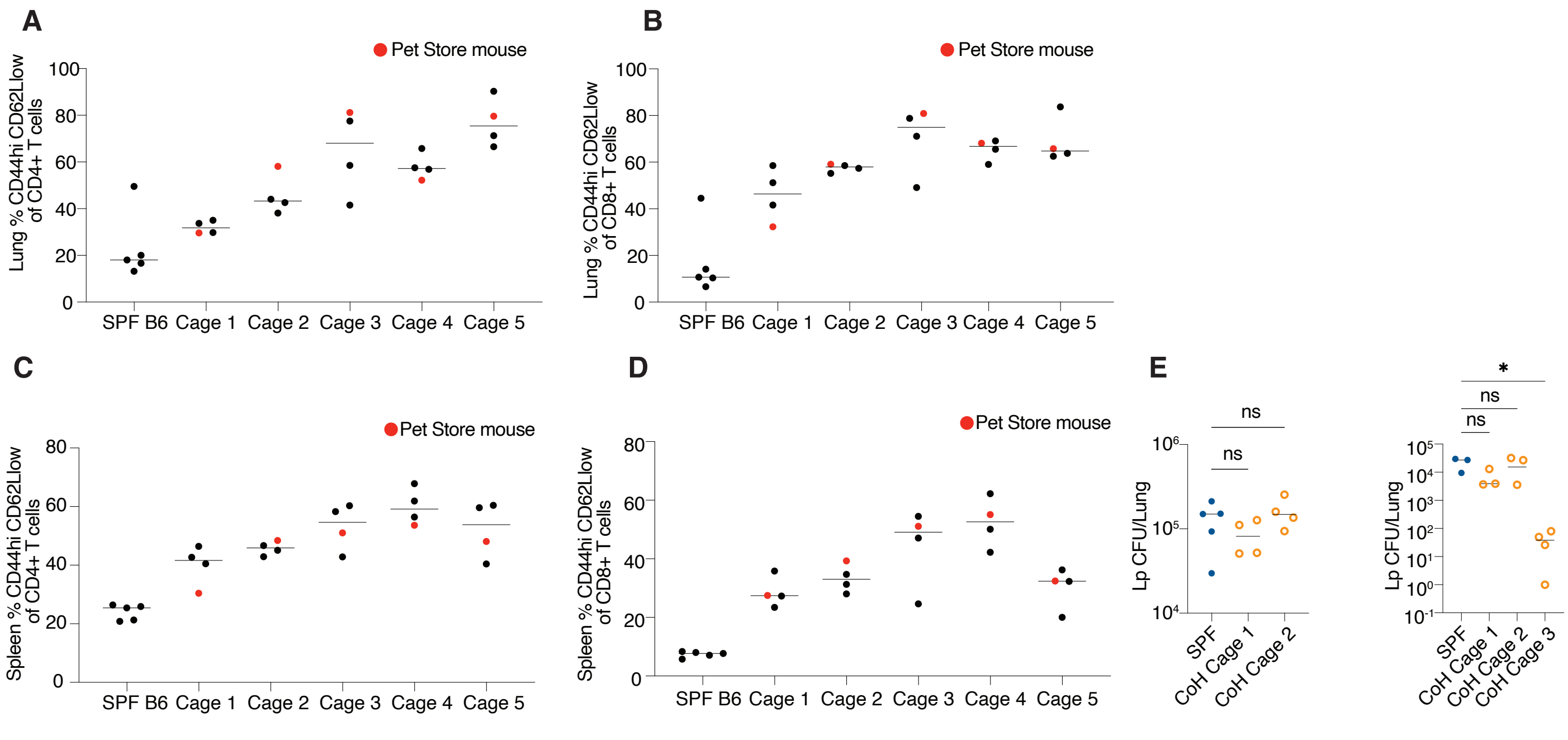

Supplementary Fig 2. The founder pet shop mouse influences cell counts and infection outcomes in cohoused C57BL/6 mice.
(**A-B**) Percent of CD4⁺ (A) or CD8⁺ (B) T effector memory cells in the lungs of either SPF C57BL/6 mice, pet shop cohoused, or pet shop mice (red). (**C-D**) % of CD4⁺ (C) or CD8⁺ (D) T effector memory cells in the spleens of either SPF C57BL/6 mice, pet shop cohoused, or pet shop mice (red). (**E**) Lp lung CFU from two separate experiments at 2 days post infection. The bars represent the median where CFU was detected. Mann-Whitney tests were used to calculate significance. *p ≤ 0.05.

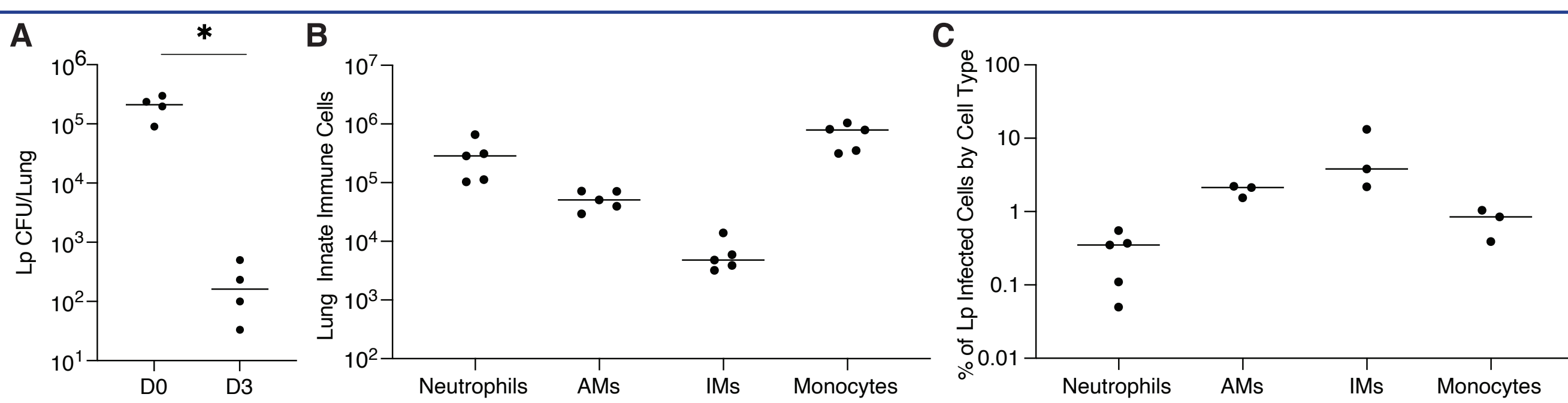

Supplementary Fig 3. Characterizing Lp infection in C57BL/6 mice.
(**A**) Lp Lung CFU measured 4 hours post infection (D0) and 3 days post-infection (D3) (**B**) Lung innate immune cells 1 day post-Lp infection. (C) Localization of Lp as measured by the detection of fluorescent bacteria as a percent of each cell type at 2 days post-infection.

bioRxiv preprint doi: https://doi.org/10.1101/2025.11.20.687493; this version posted June 11, 2026. The copyright holder for this preprint (which was not certified by peer review) is the author/funder, who has granted bioRxiv a license to display the preprint in perpetuity. It is made available under a CC-BY 4.0 International license.

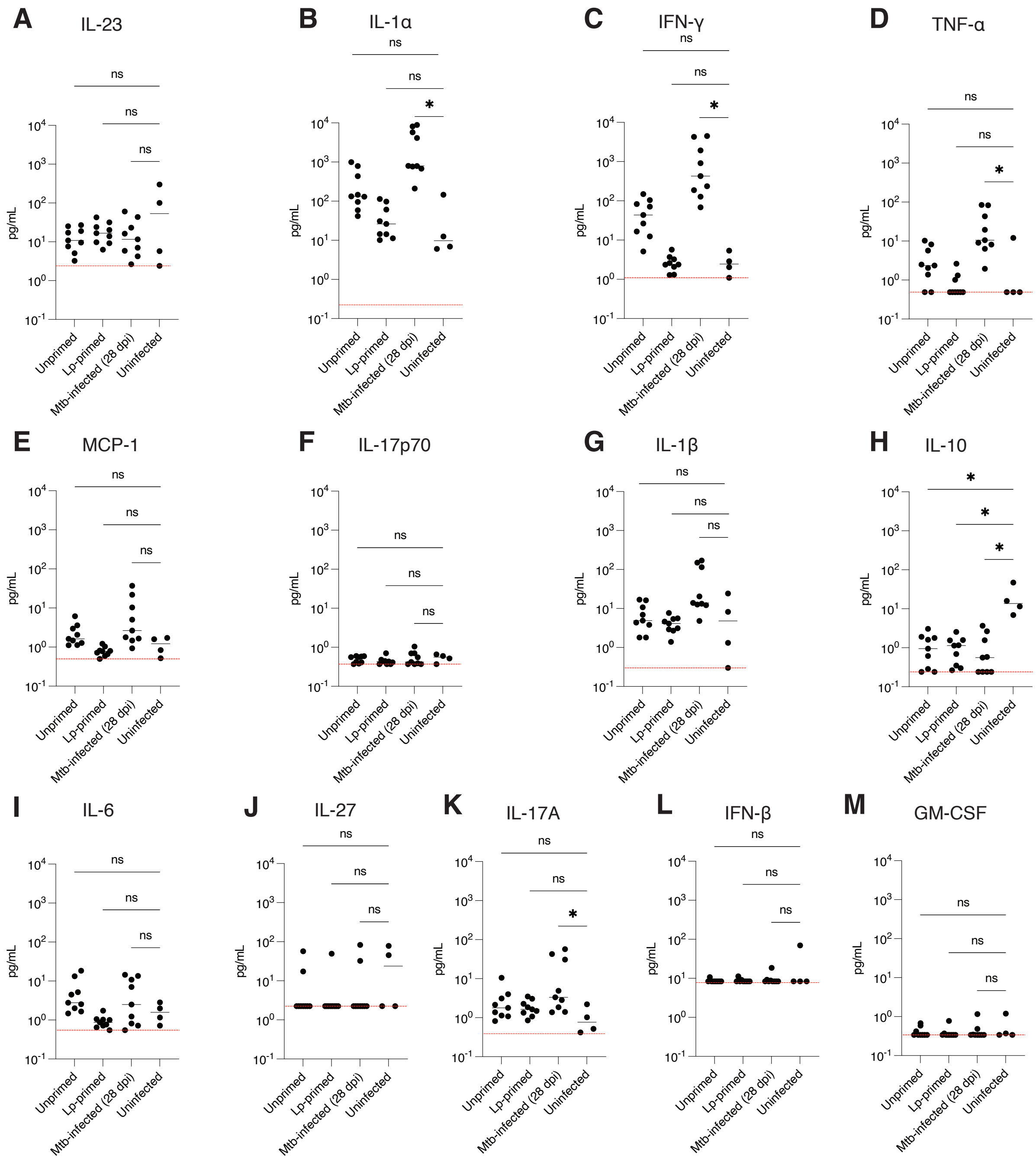

Supplementary Fig 4. Cytokine expression in the lungs of unprimed and Lp-primed C57BL/6 mice.
(**A-M**) Quantification of cytokine expression measured by LEGENDplex in unprimed or Lp-primed mice after 14 days of Mtb infection. As a positive control, mice infected with Mtb for 28 days were included. (A) IL-23, (B) IL-1α, (C) IFN-γ, (D) TNF-α, (E) MCP-1, (F) IL-17p70, (G) IL-1β, (H) IL-10, (I) IL-6, (J) IL-27, (K) IL-17A, (L) IFN-β, (M) GM-CSF. Results shown are from 2 combined experiments. Significance was calculated with a Kruskal-Wallis test followed by Dunn's multiple comparisons test. *$p \le 0.05$.

bioRxiv preprint doi: https://doi.org/10.1101/2025.11.20.687493; this version posted June 11, 2026. The copyright holder for this preprint (which was not certified by peer review) is the author/funder, who has granted bioRxiv a license to display the preprint in perpetuity. It is made available under a CC-BY 4.0 International license.

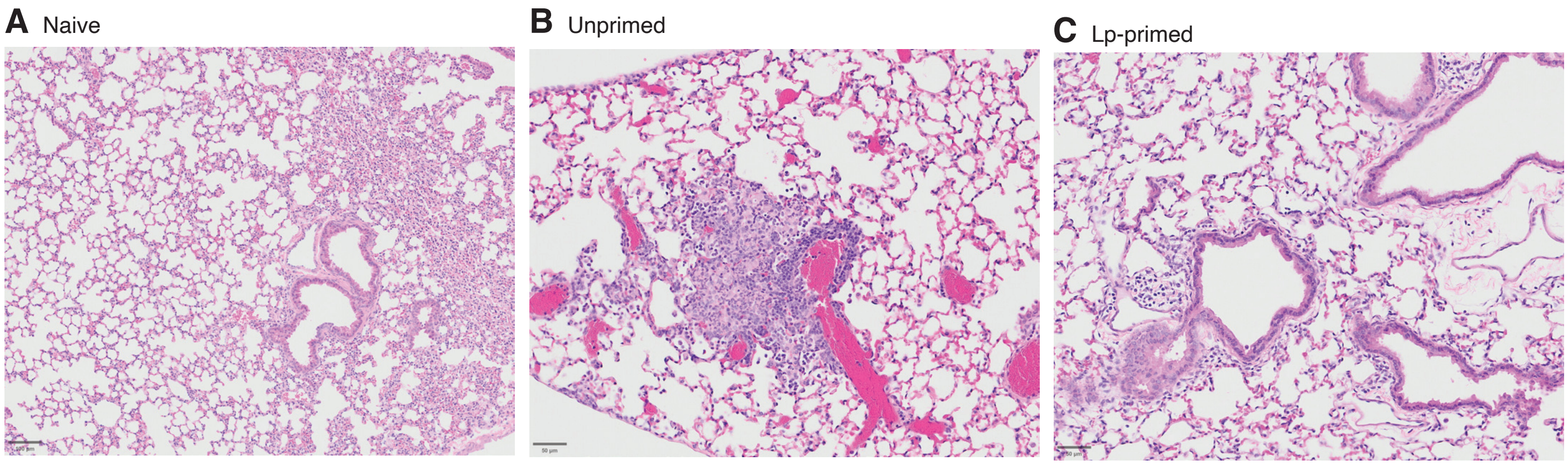

Supplementary Fig 5. Histology and acid-fast staining of lungs.
(**A-C**) Representative H&E images of either naïve, Lp-primed, or unprimed mice.

		Experiment 1			Experiment 2		
AGENTS TESTED	Methodology	Tested	Positive	Negative	Tested	Positive	Negative
Mouse Parvovirus MPV	SEROLOGY	7	3	4	7	2	5
Mouse Minute virus	SEROLOGY	7	0	7	7	4	3
Parvo NS-1	SEROLOGY	7	4	3	7	3	4
Mouse Hepatitis MHV	SEROLOGY	7	6	1	7	7	0
Murine Norovirus MNV	SEROLOGY	7	2	5	7	3	4
Theilovirus(GD7TMEV)	SEROLOGY	7	0	7	7	0	7
EDIM/Rotavirus	SEROLOGY	7	0	7	7	3	4
Sendai virus	SEROLOGY	7	0	7	7	0	7
Pneumonia virus PVM	SEROLOGY	7	0	7	7	2	5
Reovirus 1,2,3	SEROLOGY	7	0	7	7	0	7
LCMV	SEROLOGY	7	0	7	7	0	7
MAD-1 (MAV-FL)	SEROLOGY	7	0	7	7	0	7
MAD-2 (K87)	SEROLOGY	7	0	7	7	0	7
Ectromelia virus	SEROLOGY	7	0	7	7	0	7
Polyoma virus	SEROLOGY	7	0	7	7	0	7
Murine CMV	IFA	7	0	7	7	0	7
Carbacillus CARB	SEROLOGY	7	0	7	7	0	7

Supplementary Table 1. Pathogen diagnostic panel of pet shop mice.
Pathogen diagnostic panel performed on blood samples of pet shop mice by serology or immunofluorescence assay (IFA) over two independent experiments. 7 mice were tested for each experiment.

Sample	Inflammatory cell aggregation, interaveolar and septal	Inflammatory cell infiltration, perivascular and peribronchial	Vasculitis, lymphohistiocytic
Naive	-	-	-
Unprimed sample 1	Mild, multifocal, multilobe	Mild, multifocal, multilobe	Mild, multifocal
Unprimed Sample 2	Mild, multifocal, multilobe	Mild, multifocal, multilobe	Suspected, multifocal
Unprimed Sample 3	Mild, multifocal, multilobe	Mild, multifocal, multilobe	Mild, multifocal
Lp-primed Sample 1	-	Mild, multifocal, multilobe	-
Lp-primed Sample 2	Minimal (extension from perivascular infiltrate)	Mild, multifocal, multilobe	Suspected, multifocal
Lp-primed Sample 3	Minimal, focal	Minimal, multifocal, multilobe	-

Supplementary Table 2. Histological analysis of naïve, unprimed, or Lp-primed mouse lungs.
Sections of mouse lungs were stained and analyzed by a trained, blinded pathologist for signs of inflammation after 14 days of Mtb infection.

bioRxiv preprint doi: https://doi.org/10.1101/2025.11.20.687493; this version posted June 11, 2026. The copyright holder for this preprint (which was not certified by peer review) is the author/funder, who has granted bioRxiv a license to display the preprint in perpetuity. It is made available under a CC-BY 4.0 International license.

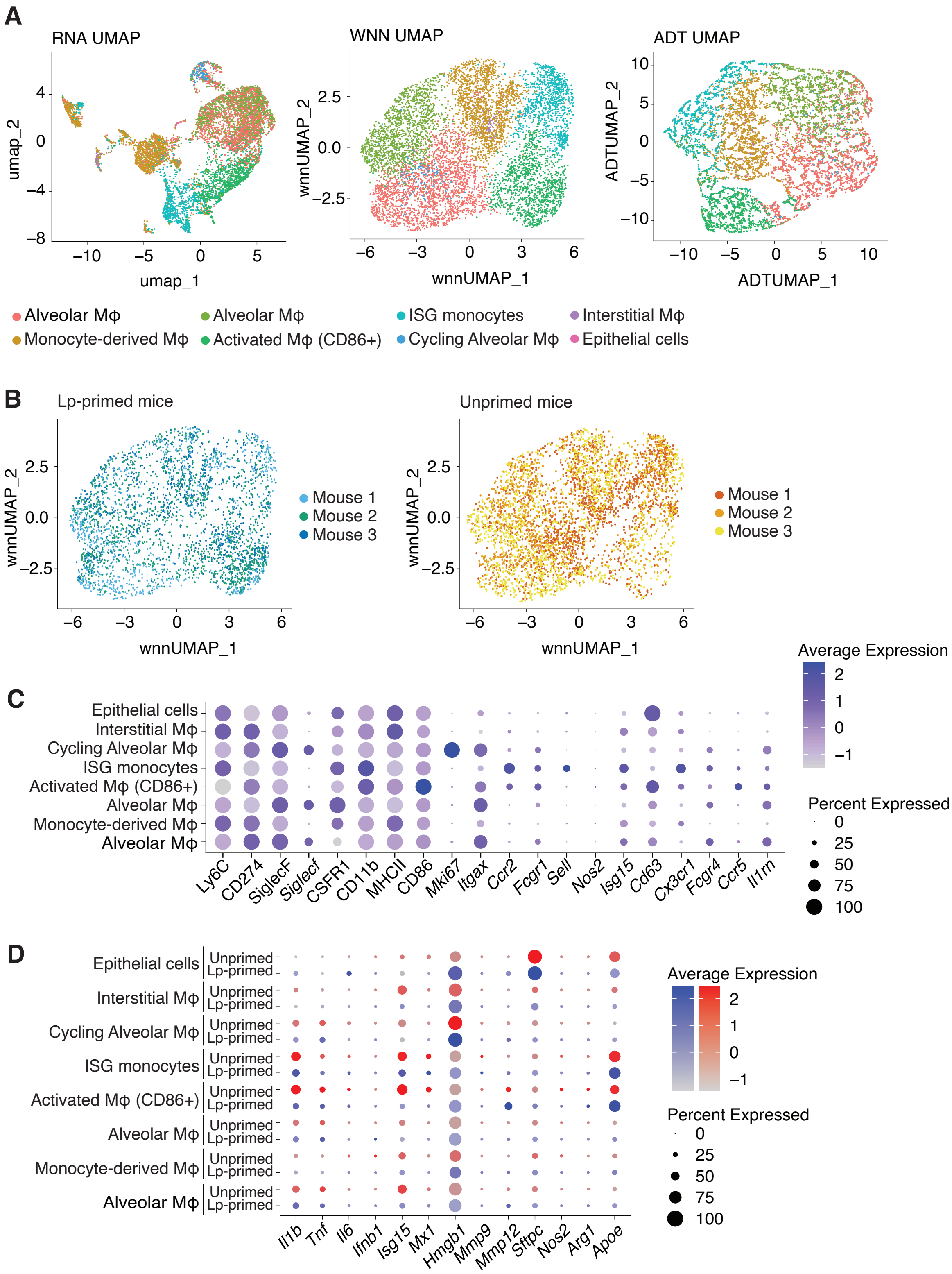

Supplementary Fig 6. Identification of lung myeloid cells and markers of activation from scRNA-seq.
(**A**) UMAP plots showing cell clustering of combined mRNA and protein expression, mRNA expression, or protein expression. Only bystander cells are shown. (**B**) UMAP plots showing cell clustering of biological replicates of unprimed or Lp-primed mice. Only bystander cells are shown. (**C**) Protein and mRNA expression of lineage-defining myeloid cell markers used for cluster classification. Both unprimed and Lp-primed mice were analyzed to identify clusters. (**D**) mRNA expression of cell state markers in unprimed and Lp-primed mice. Inflammatory mediators: *Il1b*, *Tnf*, *Il6*; Type I IFN and ISGs: *Ifnb1*, *Isg15*, *Mx1*; Macrophage activation: *Nos2*, *Arg1*, *Apoe*; Lung injury or dysfunction: *Hmgb1*, *Mmp9*, *Mmp12*, *Sftpc*.
